# Childhood imported malaria: could we have suppressed risk factors in some children?

**DOI:** 10.1186/1475-2875-11-S1-P53

**Published:** 2012-10-15

**Authors:** Selva K Pillai, Jean-Yves Siriez, Eric Kendjo, Sandrine Houze, Philippe J Guerin, Jacques Le Bras

**Affiliations:** 1 Ecole des Hautes Etudes En Santé Publique (EHESP), Paris, lle de France, 75006, France; 2 Hospital Robert-Debré APHP, Paris, Ile de France, 75019, France; 3 Centre National de Référence (CNR) du Paludisme, APHP, Paris, lie de France, 75018, France; 4 CNR Paiudisme, UMR S945 Paris 6 University, Paris, Ile de France, 75005, France; 5 Centre for Tropical Medicine, Nuffield Department of Clinical Medicine, University of Oxford, Oxford, OX3 7 LE, UK; 6 UMR 216 Paris Descartes University, Paris, Ile de France, 75006, France

## Background

Malaria infection among child travellers need special attention as it rarely occurs in non endemic countries. Most studies dealt with risks in the paediatric population, but there is a need to justify age class choices and its direct implication of analysis for severity risk factors. In this study we demonstrated that analysing the multi factorial and caregiver dependent feedbacks for children <12 and <5 years old influenced their respective severity risk factors, that was not seen when analysed as a single continuous < 18 years population.

## Materials and methods

Data collected from reported malaria cases of 2,357 children below 18 years old, from 2006-2011 to Centre National de Référence du Paiudisme, throughout Metropolitan France were analysed for historically related travel habits, demography, practices and access to medical services. Multivariate logistic regression model with best maximum likelihood and Goodness of Fit were used to assess severity outcome risks within three separate groups (0-18 years as a single population, <12 years and those <5 years). Data was analysed using STATA 11.0.

## Results

Almost 97% (n=2287) travelled the African continent, of which 94% (n=2216) to African countries historically-linked with France. There were 101 severe cases with a case fatality ratio of 0.17% over the study period. The 0-18 years group analysis revealed children of expats/residents >6months had severity risks of OR 3.4 (95% CI: 1.39-8.30) and being born in endemic countries confirmed a protective factor against severity in all 3 groups analysed. However, evaluation of <12 years old, surfaced the risk of severity associated with declaration of chemoprophylaxis intake with OR 3.08 (95%CI: 1.31-7.24), thus not fulfilling its protective role. Declaration of inappropriate doxycycline use <12 years old was also detected. Being <5 years old and being born in France conferred a protective effect of OR 0.15 (95% CI: 0.02-0.97) were both reassuring and a new contribution in understanding imported childhood malaria. Medical services were promptly used by caregivers in France with diagnostic services and treatment initiated within the same day (mean 0.07 days, 95% CI: 0.05-0.95). Caregivers’ response accuracy of providing date of symptoms onset, were validated through an innovative approach.

## Conclusion

By improvising approach, we revealed both exclusive risk and protective factors for children <12 and <5 years old. Despite the presence of awareness among the caregivers to provide chemoprophylaxis to their children, possibility of unregulated or inappropriate chemoprophylaxis intake among child travellers became a risk factor for severe malaria, as it provided a false sense of security. Effective preventive medicine must include regulating chemoprophylaxis and community practices of them. Young children <5 were less likely to develop imported severe malaria. We hypothesized that having easy access and frequent contact to a good healthcare system and being young infants of possibly new immigrant mothers with immunity may partially explain this finding. For effective risk reduction, respect must be given to the unique age, developmental stage and characteristic of the paediatric population. Travel habits to countries historically-linked with France should be explored and leveraged to effectively reduce severe imported and endemic childhood malaria.

